# Tele‐medicine and improvement of mental health problems in COVID‐19 pandemic: A systematic review

**DOI:** 10.1002/mpr.1924

**Published:** 2022-06-14

**Authors:** Hossein Hatami, Niloofar Deravi, Bardia Danaei, Moein Zangiabadian, Amir Hashem Shahidi Bonjar, Ali kheradmand, Mohammad Javad Nasiri

**Affiliations:** ^1^ Department of Public Health School of Public Health and Safety and Environmental and Occupational Hazards Control Research Center Shahid Beheshti University of Medical Sciences Tehran Iran; ^2^ Department of Microbiology School of Medicine Shahid Beheshti University of Medical Sciences Tehran Iran; ^3^ School of Dentistry Clinician Scientist of Dental Materials and Restorative Dentistry Shahid Beheshti University of Medical Sciences Tehran Iran; ^4^ Department of Psychiatry Taleghani Hospital Research Development Committee Shahid Beheshti University of Medical Sciences Tehran Iran

**Keywords:** COVID‐19, mental disorder, SARS‐CoV‐2, tele‐medicine

## Abstract

**Introduction:**

Tele‐medicine services have been developed in response to the COVID‐19 pandemic, which disrupts mental health services. The present study investigates the effectiveness of telephone‐delivered services for psychological disorders in the COVID‐19 pandemic.

**Methods:**

We searched PubMed/Medline, Embase and Cochrane Controlled Register of Trials for relevant clinical studies up to February 1, 2022. Following terms were used: “severe acute respiratory syndrome”, “Coronavirus”, “Coronavirus infection”, “SARS‐CoV‐2”, “COVID‐19”, “mental disorder”, “mental health”, “mental health program”, “mental health service”, “psychiatric service”, “telemedicine”, “Telehealth”, “Tele‐health”, “Telecare”, “Mobile health”.

**Results:**

Twelve relevant clinical articles were included in our study. Eight articles were parallel randomized controlled trials (RCTs), two were Quasi‐experimental, and one was a multicenter retrospective cohort study. A total of 1900 adults (18 years old or above that) were included. Online telecommunication methods like online apps and videoconference were the most common interventions. The most prevalent measured outcome was levels of anxiety and depression among participants. Eleven out of 12 articles showed a significant association between telemedicine and mental health improvement.

**Conclusions:**

The included studies in the current systematic review reported the probable efficacy of telemedicine in improving mental health disorders during the COVID‐19 pandemic. But it is not possible to determine the best telecommunication method for each mental disorder in different populations and the preference of patients is still face to face therapy. So RCTs in different populations with previous mental disorders or chronic diseases are required to investigate the further telemedicine's efficacy on managing mental problems.

## 1 INTRODUCTION

An outbreak of the novel coronavirus disease (COVID‐19) occurred at the end of 2019. Since then, COVID‐19 has spread rapidly throughout the world. Mortality rates appeared to be about 2%, with an additional 15%–20% requiring hospitalization. Currently, treatment is mainly supportive. The virus is highly contagious and therefore can spread rapidly.

The COVID‐19 crisis is multidimensional, with impacts across functional dimensions, including emotionally, economically, physically, psychologically, and socially. Although anxiety and fear should be normalized during this time, it is still essential to understand how these can be exacerbated due to uncertainty, social distancing, and economic downfall (Sullivan et al., 2020). A significant concern is that during the current pandemic, the mentioned exacerbations and limited access to care may worsen psychiatric illnesses. Furthermore, quarantine can mean losing freedom, separation from loved ones, and uncertainty regarding health status. Various studies suggest that when individuals are in social isolation or quarantined for different purposes, the psychological impact would be substantial, wide‐ranging, and long‐lasting. These concerns may have considerable effects on mental health status (Brooks et al., 2020; Xiang et al., 2020) for both patients and health care providers (Lai, Ma, et al., 2020). Moreover, social isolation and loneliness are linked to worsening depression and anxiety and a significantly increased risk of hospitalization (Stephenson, 2020). However, with the help of telemedicine, this burden could be reduced because of its convenience during this pandemic situation (Zhou et al., 2020).

Telehealth, defined as the delivery of psychological and mental health services via telecommunication technologies, has been previously described as ‘the next big frontier in the efficient and effective delivery of health care (Varker et al., 2019). Its modalities include videoconferencing, telephone‐delivered therapy, internet‐delivered programs, and mental health apps (Nelson et al., 2011). The advantages of telemedicine in the current pandemic situation have been discussed with several controversies. Telemedicine can support health administration, long‐distance clinical care, and education. The patients seeking care for depression and anxiety could be assisted without visiting a hospital. Without the typical face‐to‐face visit with the doctor, therapy for psychological stabilization could be provided. Moreover, telemedicine can reduce the loss of follow‐up among psychiatric patients, and by reducing the number of clinic visits for medicines and periodical discussions among the geriatric populace with mental ailments, telemedicine can also possibly decrease the number of secondary or tertiary diseases (Hau et al., 2020).

On the other hand, its execution has several challenges, including a breakdown in the relationship between health professionals, issues about the quality of health information data, and bureaucratic and organizational challenges (Hjelm, 2005). Also, there are several ethical and legal issues related to the practice of these telehealth services, like informed consent and autonomy, patient privacy and confidentiality, data protection and security, equity of access, etc (Solimini et al., 2021). So there are limited data regarding the efficacy of telemedicine for the treatment of mental disorders during the COVID‐19 pandemic. Therefore, this systematic review aims to investigate the association of telemedicine and the improvement of mental health problems.

## 2 METHODS

This systematic review conforms to the “Preferred Reporting Items for Systematic Reviews and Meta‐Analyses” (PRISMA) statement (Institute, 2017).

### 2.1 Search strategy

The English medical literature search was carried out in PubMed/Medline, Embase and the Cochrane Controlled Register of Trials (CENTRAL) up to February 1, 2022. Clinical studies investigating the relationship between mental health issues due to the COVID‐19 pandemic and psychological interventions were selected.

We used the following terms: “severe acute respiratory syndrome”, “Coronavirus”, “Coronavirus infection”, “SARS‐CoV‐2”, “COVID‐19”, “mental disorder”, “mental health”, “mental health program”, “mental health service”, “psychiatric service”, “telemedicine”, “Telehealth”, “Tele‐health”, “Telecare”, “Mobile health” and similar terms which are attached in the appendix in detail.

### 2.2 Study selection

The records found through database searching were merged, and the duplicates were removed using EndNote X7 (Thomson Reuters). Two reviewers independently screened the records by title/abstract and full text to exclude those unrelated to the study topic. Included studies met the following criteria: 1) Study population were individuals susceptible to mood and anxiety disorders such as depression or generalized anxiety disorder (GAD) in the basis of self‐reporting questionnaires or academic scales and scores (like PHQ‐9 questionnaire or GAD‐7 scale) due to the COVID‐19 pandemic and its consequences on life style; 2) Intervention and observation duration of at least 2 weeks; 3) Participants of at least 18 years or older.

The primary outcome assessed was the improvement of psychological and mental problems, such as loneliness, depression symptoms, and stress levels among participants. Conference abstracts, editorials, and reviews, were excluded.

### 2.3 Quality assessment

Two reviewers, Bardia Danaei and Niloofar Deravi, assessed the studies' quality using two different assessment tools; The Newcastle‐Ottawa Scale (NOS) for observational studies and the cochrane tool for experimental studies (Higgins et al., 2011; WellsGA et al., 2012). Third reviewer Mohammad Javad Nasiri was planned to decide if the two reviews couldn't agree on a particular point of bias assessment.

The NOS scale evaluates the risk of bias of prospective studies with three domains: (1) selection of participants, (2) comparability, and (3) outcomes. A study can be awarded a maximum of one point for each numbered item within the selection and outcome categories. A maximum of two points can be given for comparability. Scores of 0–3, 4–6, and 7–9 were assigned for the low, moderate, and high‐quality studies, respectively. The Cochrane tool is based on; the use of random sequence generation; concealment of allocation to conditions; blinding of participant and personnel; blinding of outcome assessors; completeness of outcome data and other; selective reporting and other biases. Each study was rated as low risk of bias when there was no concern regarding bias; as high risk of bias when there was concern regarding bias; or unclear risk of bias if the information was absent.

### 2.4 Data extraction

For each study, variables such as first author, nationality, gender, age, type of study, type of interventions, and the final outcome were extracted. Two authors were involved in data extraction, and disagreements were discussed, and if applicable, another reviewer's opinion was asked.

## 3 RESULTS

**FIGURE 1:**
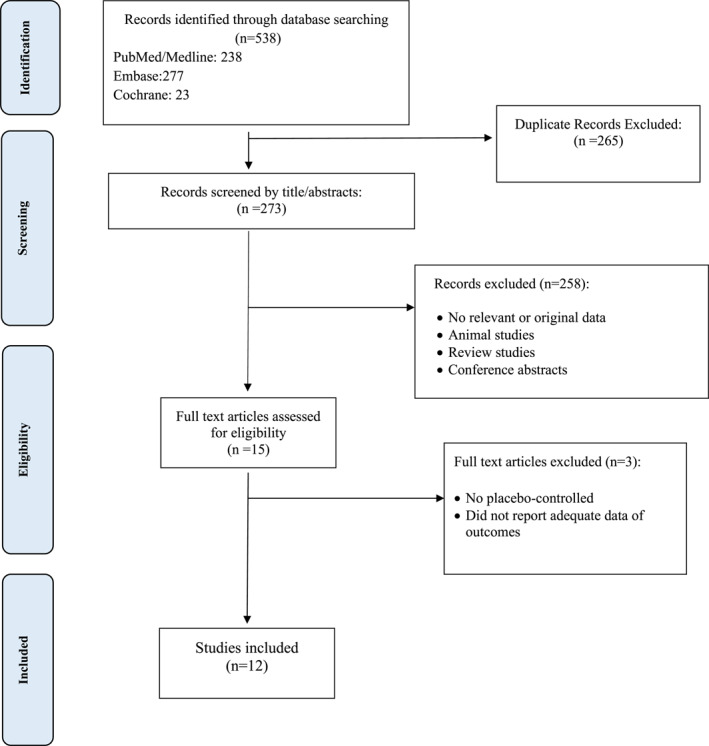
Flow chart of the number of studies identified and selected into the systematic review

Twelve articles were included via the selection process, shown in Figure 1. Nine articles were parallel randomized controlled trials (RCTs), two were Quasi‐experimental, and one was a multicenter retrospective cohort study. Three of these studies were conducted in the USA, two in Spain, and others in Brazil, Oman, Canada, Hong Kong, Israel, Netherlands, and United Kingdom. The population of these articles consists of approximately 1900 adults (18 years old or above that). Participants were individuals prone to mental illnesses like depression and GAD due to the COVID‐19 pandemic and its consequences on lifestyle. Interventions in these studies consisted of a wide range of telecommunication methods, including telephone contacts (Alessi et al., 2021, 2022; Kahlon et al., 2021; Lai, Yan, et al., 2020), messaging platforms (Aguilera et al., 2021), online methods, for example, videoconference, and online apps (Al‐Alawi et al., 2021; Fiol‐DeRoque et al., 2021; Lai, Yan, et al., 2020; Sanchez‐Guarnido et al., 2021; Shapira et al., 2021; Summers et al., 2021; Watts et al., 2020; Weerkamp‐Bartholomeus et al., 2020). The outcome assessment was different between the articles. These articles reported a wide range of psychological and mental problems, including emotional distress, anxiety, depression, loneliness, eating or sleeping disturbances, negative affect and also, self‐efficacy, quality of life, and working alliance as their outcomes, but the most commonly measured outcome was the rate of anxiety and depression (Table 1).

**TABLE 1:** The association of telemedicine on the improvement of mental health problems

Authors	Year	Country	Type of study	Participants' age category	Intervention type	Outcomes	Association of intervention on outcome
Alessi et al., 2021	2021	Brazil	Parallel RCT	Adult	Telephone contacts and providing educational materials on issues related to mental health and healthy habits	Emotional distress	Yes
Fiol‐DeRoque et al., 2021	2021	Spain	Parallel RCT	Adult	An app targeting emotional skills, healthy lifestyle behavior, burnout, and social support with psychotherapy	Depression, anxiety, stress, insomnia, burnout, posttraumatic stress, self‐efficacy	No
Kahlon et al., 2021	2020	USA	Parallel RCT	Adult	Telephone contacts and empathetic conversations	Loneliness, depression, anxiety, mental health	Yes
Al‐Alawi et al., 2021	2021	Oman	Parallel RCT	Adult	Online sessions utilizing cognitive behavioral therapy and acceptance and commitment therapy	Anxiety, depression	Yes
Watts et al., 2020	2019	Canada	Parallel RCT	Adult	Tele psychotherapy via videoconference	Working alliance	Yes
Lai, Yan, et al., 2020	2020	Hong Kong	Parallel RCT	Adult	Telephone contacts and video communication providing educational materials and health services	Neurocognitive function, behavioral and psychological problems, quality of life, mental health	Yes
Shapira et al., 2021	2020	Israel	Parallel RCT	Adult	Online guided group sessions	Loneliness, depression	Yes
Weerkamp‐Bartholomeus et al., 2020	2020	Netherlands	Parallel RCT	Adult	Online wiring affect with reattach therapy	Negative affect	Yes^a^
Alessi et al., 2022	2021	Brazil	Parallel RCT	Adult	Psychiatric and health consultation via telephone contacts	Emotional distress	Yes
Summers et al., 2021	2021	United Kingdom	Quasi experimental	Adult	An app that provides behavioral change support through education and guided activities about issues related to mental health and healthy habits	Anxiety, depression	Yes
Sanchez‐Guarnido et al., 2021	2021	Spain	Retrospective cohort	Adult	Occupational intervention via telehealth and telephone	Hospital admissions and relapses due to mental illness, depression	Yes
Aguilera et al., 2021	2021	USA	Quasi experimental	Adult	An automated text messaging platform sending skills‐based and mood messages	Anxiety, depression	Yes

Abbreviation: RCT, randomized controlled trials.

^a^ There was a significant decrease in negative affect after W.A.R.A. remote therapy compared to the negative affect before the intervention (*p* < 0.001) but the reduction of negative affect was larger after face‐to‐face therapy than after remote therapy (*p* < 0.001).

### 3.1 Quality of included studies

Quality of included studies was assessed. Based on the NOS, which was used to evaluate the quality of the only observational study, the mean (standard deviation [SD]) NOS score was 8.0, which is suggestive for high methodological quality and a low risk of bias of the included studies. More detailed information about the quality assessment of the observational studies can be seen in Table 2.

**TABLE 2:** Quality assessment of the observational studies included in the meta‐analysis (The Newcastle‐Ottawa Scale (NOS) tool)

Author	Selection				Comparability		Outcome			
	Representativeness of exposed cohort	Selection of non‐exposed cohort	Ascertainment of exposure	Demonstration that outcome of interest was not present at start of study	Adjust for the most important risk factors	Adjust for other risk factors	Assessment of outcome	Follow‐up length	Loss to follow‐uprate	Total quality score
Sánchez‐Guarnido (20)	1	1	1	1	1	0	1	1	1	8

The risk of bias assessment of the experimental studies according to the Cochrane tool is presented in Table 3. Only two studies (Aguilera et al., 2021; Summers et al., 2021) have a high risk of bias in the cases of allocation concealment, blinding of participants, and blinding of outcome (Table 3).

**TABLE 3:** Quality assessment of the experimental studies included in the meta‐analysis (the Cochrane tool)

Author	Random sequence generation	Allocation concealment	Blinding of participants and personnel	Blinding of outcome assessment	Incomplete outcome data	Selective reporting	Other bias
Alessi et al., 2021	Low risk	Low risk	Low risk	Low risk	Low risk	Low risk	Low risk
Fiol‐DeRoque et al., 2021	Low risk	Low risk	Low risk	Low risk	Low risk	Low risk	Low risk
Kahlon et al., 2021	Low risk	Low risk	Low risk	Low risk	Low risk	Low risk	Low risk
Al‐Alawi et al., 2021	Low risk	Low risk	Low risk	Low risk	Low risk	Low risk	Low risk
Watts et al., 2020	Low risk	Low risk	Low risk	Low risk	Low risk	Low risk	Low risk
Lai, Yan, et al., 2020	Low risk	Low risk	Low risk	Low risk	Low risk	Low risk	Low risk
Shapira et al., 2021	Low risk	Low risk	Low risk	Low risk	Low risk	Low risk	Low risk
Weerkamp‐Bartholomeus et al., 2020	Low risk	Low risk	Low risk	Low risk	Low risk	Low risk	Low risk
Alessi et al., 2022	Low risk	Low risk	Low risk	Low risk	Low risk	Low risk	Low risk
Summers et al., 2021	High risk	High risk	High risk	Low risk	Low risk	Low risk	Low risk
Aguilera (17)	High risk	High risk	High risk	Low risk	Low risk	Low risk	Low risk

### 3.2 The association of telemedicine on the improvement of mental health problems

In the study conducted by Alessi et al., 91 adults aged 18 or older with a previous diagnosis of type 2 diabetes and no mental illness were included. Forty‐six adults in the intervention group received telephone contacts and educational materials on issues related to mental health, healthy habits, and diabetes care in 16 weeks during the COVID‐19 pandemic. The primary outcome was a positive screening for mental health disorder with the help of a self‐reporting questionnaire after intervention, and the secondary outcome included a positive screening for diabetes‐related emotional distress, eating and sleep disorders. After analyzing, a positive screening for mental health disorders was found in 37% of participants in the intervention group versus 57.8% in the control group (*p* = 0.04). The rate of diabetes‐related emotional distress was significantly higher in the control group than the intervention group (*p* = 0.03). But there was no significant difference regarding eating and sleep disorders (Alessi et al., 2021).

In the study conducted by Fiol‐DeRoque et al., 482 health care workers providing face‐to‐face health care to patients with COVID‐19 were included. The intervention group consisted of 248 healthcare workers who received the Psycovid app intervention for 2 weeks. Psycovid app is an application targeting emotional skills, healthy lifestyle behavior, burnout, and social support. The primary outcome of this study was a composite of depression, anxiety, and stress. The secondary outcome were insomnia, burnout, posttraumatic stress, and self‐efficacy. There were no significant differences between the two groups at 2 weeks in the primary or other outcomes. Still, there were substantial improvements among health care workers consuming psychotropic medications (*p* = 0.02) or receiving psychotherapy (*p* = 0.004) (Fiol‐DeRoque et al., 2021).

Kahlon et al. conducted a parallel randomized clinical trial on the Meals on Wheels Central Texas clients, at‐risk older adults. Two hundred 40 healthy adults with a mean age of 69.05 were included. One hundred 20 adults were in the intervention group and received calls from callers briefly trained in empathetic conversational techniques for 4 weeks. The primary outcome was loneliness, and the secondary outcomes were depression, anxiety, and self‐related health. The results show significant improvement in loneliness (*p* < 0.001), depression (*p* < 0.001), general anxiety disorder (*p* < 0.001), and overall mental health (*p* = 0.003) (Kahlon et al., 2021).

In the study conducted by Al‐Alawi et al., 60 participants were recruited from a study sample surveyed for symptoms of anxiety or depression among the Omani public amid the COVID‐19 pandemic. Twenty‐two of them participated in weekly online sessions from certified psychotherapists who utilized cognitive behavioral therapy and acceptance and commitment therapy interventions for 6 weeks. The primary outcome was the change in the mean scores of Patient Health Questionnaire‐9 (PHQ‐9), and General Anxiety Disorder‐7 (GAD‐7) scale from the baseline to the end of the study (i.e., after six sessions) between the two groups, and the secondary outcome was to compare the proportions of participants with depression and anxiety between the two groups. The reduction was significant in GAD‐7 (*p* = 0.01) and the intervention group's PHQ‐9 scores (*p* = 0.006). Also, reduction of anxiety and depression was higher in the intervention group (*p* = 0.049) than in the control group (*p* = 0.02) (Al‐Alawi et al., 2021).

In another study conducted by Watts et al., 115 participants were recruited from university‐based psychology clinics specializing in anxiety disorders across five cities in the Province of Québec (Canada). Sixty‐nine of them received telepsychotherapy via videoconference, and others underwent conventional psychotherapy for 15 weeks. The measured outcome was the global working alliance inventory score difference between controls and subjects. The aggregated global working alliance inventory score analysis revealed a significant clinical and statistical improvement of the participants in both groups but did not showed a significant difference between the two treatment conditions (Watts et al., 2020).

Ho‐yin Lai conducted a study on 60 community‐dwelling people with cognitive impairment and their spousal caregivers. Half of the participants were in the intervention group. Both arms of the study received weekly 30 min care service via telephone covering information relevant to older adults for 4 weeks. Still, in addition to that, the intervention arm received 30 min of health services delivered through communication apps. The primary outcomes were quality of life, cognitive, memory, and behavior assessment, and the secondary outcomes were physical and mental health status, caregiver burden based on feelings of over‐sacrifice, perceived care‐recipient's dependence, negative emotions during care, feelings of inadequacy, and uncertainty about the care‐recipient's future and self‐efficacy of caregivers. In all the measured parameters, there was a significant improvement in the intervention group compared to the control group (Lai, Yan, et al., 2020).

In the study conducted by Shapir et al., participants were 86 community‐dwelling adults aged 65 and older. Sixty‐eight individuals took part in twice‐weekly online guided group sessions via an application in 4 weeks. The moderators were clinical social workers who underwent designated training by a senior clinical social worker from the research team. The outcome measured was loneliness and depressive symptoms. The results showed a significant reduction in loneliness (*p* = 0.02) and depressive symptoms (*p* = 0.05) (Shapira et al., 2021).

In a study by Weerkamp‐Bartholomeus et al., there were 83 patients with stress‐related complaints which 37 of them were in the intervention group received remote Wiring Affect with Re‐Attach (W.A.R.A.) therapy. In contrast, the control group received face‐to‐face W.A.R.A. therapy. The results showed that the remote treatment reduced negative effects among participants significantly (*p* < 0.001), but the reduction was more considerable after W.A.R.A. face‐to‐face than after W.A.R.A. remote therapy (*p* < 0.001) (Weerkamp‐Bartholomeus et al., 2020).

In another RCT study conducted by Alessi et al., 58 individuals with a previous diagnosis of type 1 diabetes with regular follow‐up in two public care centers in Southern Brazil were selected. Twenty‐nine participants were in the intervention group that received weekly telephone contacts with the appointment protocol prepared by a multidisciplinary team that lasted 16 weeks. The results showed a significant decline in emotional distress (*p* = 0.43) (Alessi et al., 2022).

In the quasi‐experimental study conducted by Summers et al., 273 adults who had joined the Gro health app participated. Gro Health is a digital health intervention that provides behavioral change support through structured education and guided activities in mental well‐being, nutrition, sleep, and exercise. The intervention lasted 12 weeks, and the changes in scores for anxiety, perceived stress, and depression were measured. The results showed a significant reduction in depression (*p* < 0.001), Anxiety (*p* < 0.001), and stress (*p* < 0.001) among participants after the intervention (Summers et al., 2021).

In a multicenter retrospective cohort study conducted by Sánchez‐Guarnido et al., 270 patients with mental disorders diagnosed under follow‐up in day hospitals during 2020 were included. This study compared occupational intervention via telehealth to face‐to‐face occupational therapy. The outcome was hospital admissions due to their mental illness and relapses. At 2 months, the percentage of patients admitted was significantly lower in the telehealth intervention group compared to the non‐intervention group. These differences were maintained at four (*p* = 0.007) and 6 months (*p* = 0.001) (Sanchez‐Guarnido et al., 2021).

In another quasi‐experimental study conducted by Aguilera et al., 193 Healthy individuals were enrolled in the StayWell trial using the Healthy Short Message Service (SMS) platform. Participants received one daily skills‐based message and one mood message that did not vary for 60 days. This paper described the changes in StayWell participants' anxiety and depression levels after 60 days of exposure. The study's results showed statistically significant reductions in both depression and anxiety scores from baseline (*p* < 0.001) (Aguilera et al., 2021).

## 4 DISCUSSION

In the present study, we showed the probable efficacy of different types of telemedicine in varied population. Eleven out of 12 studies represented the significant effect of telemedicine on different aspects of mental health like emotional distress, depression, anxiety, etc. But there were heterogeneity in sample size, target population, telecommunication methods, measurements and outcomes so it is difficult to determine an obvious effect of a specific telemedicine tool on a specific mental disorder.

With the rapid worldwide spread of the COVID‐19 pandemic, social systems were forced to adapt to a changing society, characterized by working from home, physical distancing, and increased levels of fear and uncertainty (Greenhalgh et al., 2020; Wind et al., 2020). The pandemic catalyzed the changes in the delivery of various health care services. Researchers worldwide study remote delivery of psychological treatment services; studies conducted before the pandemic have also reported that the efficacy of remote‐based treatment services could be as high as that of the face‐to‐face programs (Wright & Caudill, 2020; Wright et al., 2019).

Telehealth interventions could be described as synchronous or asynchronous (Cooper et al., 2020). Synchronous treatment options are interactive communications that occur in real‐time, like telephone and video conferencing, and are the most similar to face‐to‐face treatment. On the other hand, asynchronous treatments include text, apps, faxes, emails, and online programs. Some practitioners already use asynchronous intervention options to check on patient's progress, online assessments provide supplementary materials, and recommend online programs or mental health apps (Cooper et al., 2020). In our study, seven articles had used synchronous interventions (call or video conferecing) and all of them revealed significant effect. The only study that had not significant effect had used asynchronous intervention (Psycovid app).

An RCT by Al‐Alawi focusing on COVID‐19–induced symptoms of anxiety and depression comparatively assessed the efficacy of therapist‐guided online therapies with that of self‐help, internet‐based treatments. Accordingly, compared to the self‐help group, therapist‐guided online therapies resulted in a significantly more significant reduction of depression and anxiety. These results can support the idea that self‐help materials and online medicines are functionally distinct (Bennett et al., 2019). A telephone‐based, empathy‐focused program conducted in 2020 was also reported to reduce loneliness, anxiety, and depression, in homebound adults who required meals from a community‐based provider (Kahlon et al., 2021). Other RCTs have also supported the use of synchronous interventions such as Telephone contacts and video communication providing educational materials and health services in the pandemic of COVID‐19 (Lai, Yan, et al., 2020; Shapira et al., 2021).

A recent trial evaluated the effectiveness of a psychoeducational mobile Health (mHealth) intervention to reduce mental health problems in healthcare workers during the COVID‐19 pandemic. It observed that the intervention could not produce significant effects among healthcare workers using the intervention in the absence of any additional mental help. However, PsyCOVIDApp effectively improved outcomes when used in conjunction with evidence‐based treatments (like psychotherapy and psychotropic medications) (Fiol‐DeRoque et al., 2021). This finding is also in line with results from a recent systematic review, reporting a lack of effect of various mental mHealth interventions when used as a standalone therapy (Weisel et al., 2019). Also, it is essential to consider that the duration of intervention in this trial was 2 weeks which was the shortest among other studies included in this paper (Fiol‐DeRoque et al., 2021).

Some populations are more vulnerable to the psychosocial effects of pandemics (Pfefferbaum & North, 2020). Under non‐pandemic conditions, patients with diabetes mellitus generally have higher mood and anxiety disorders rates than the general population (Meurs et al., 2016), so it was expected that they might be affected more significantly under the pandemic situation. A recent study published by Alessi et al. showed 44.2% of the prevalence of minor psychiatric disorders in patients with diabetes during the COVID‐19 pandemic (Alessi et al., 2020). In another study by Alessi et al. (2021), at the end of 4 months of the pandemic, almost 60% of patients with diabetes had a positive screening result for mental health disorders. It was possible to reduce this number to 36% by maintaining regular phone calls with health care specialists. The benefits of contact reduced diabetes‐related emotional distress as well. These data reinforce the importance of developing remote care strategies to mitigate the psychological effects of the COVID‐19 pandemic, especially for patients with type 2 diabetes.

According to Watts et al. (2020) study on the working alliance, psychotherapists claimed there is no difference in the quality of the therapeutic relationship they developed with their clients, regardless of the treatment condition (telepsychotherapy via video conference or conventional psychotherapy). This result would question the notion that psychotherapists are systematically biased against telepsychotherapy, leading to underestimating the potential quality of the relationship developed using this communication medium. Also, the recent systematic review and meta‐analysis has represented the similarity of working alliance along with symptom severity, overall improvement, function and client satisfaction in both telehealth and face to face psychotherapy (Greenwood et al., 2022). On the contrary, in Weerkamp‐Bartholomeus et al. study (Weerkamp‐Bartholomeus et al., 2020), although the remote therapy significantly reduced the negative affect but they showed the preference of face to face therapy. This result is in line with findings of a RCT of 325 Chicago‐area primary care patients with major depressive disorder who received Telephone‐administered Cognitive Behavioral Therapy (T‐CBT) versus face to face Cognitive Behavioral Therapy (CBT) and it was revealed that although Patients showed significant improvement in depression across both treatments but those receiving face‐to‐face CBT were less depressed than those receiving T‐CBT (Mohr et al., 2012). The reason of this preference may be because of the intrinsic barriers of telemedicine such as lack of eye contact as well as physical and social contact (poor body language and communication), patients' difficulty in expressing emotions, patients' lack of seriousness and technological incompatibility (Almathami et al., 2020).

Our systematic review holds some limitations. Firstly, some of the interventional methods used in selected articles was based on modern technology which can create some challenges for the elderly, which makes the implementation of this methods more difficult in this age group. Secondly, the limited number of participants and institutions and the duration of studies were obstacles that need to be noticed. The studies also had different settings and methodologies. Likewise, due to the limited number of studies, potential factors that lead to overall heterogeneity were not examined. Finally, the current systematic review did not examine telemedicine practices occurring pre‐pandemic.

In conclusion, the included studies in the current systematic review reported the probable efficacy of telemedicine in improving mental health disorders during the COVID‐19 pandemic. But it is not possible to determine the best telecommunication method for each mental disorder in different populations and the preference of patients is still face to face therapy. So RCTs in different populations with previous mental disorders or chronic diseases are required to investigate the further telemedicine's efficacy on managing mental problems.

## AUTHOR CONTRIBUTIONS

All authors contributed to the article and approved the submitted version.

## CONFLICT OF INTEREST

The authors declare no conflict of interest.

## Data Availability

The data that support the findings of this study are available from the corresponding author upon reasonable request.

## Appendix

Pubmed/Medline:

(SARS[tiab] OR "severe acute respiratory syndrome"[tiab] OR Coronavirus[tiab] OR "Coronavirus infection"[tiab] OR COVID[tiab] OR COVID‐19[tiab] OR 2019‐nCoV[tiab] OR SARS‐Cov‐2[tiab] OR "Novel coronavirus"[tiab] OR 2019‐nCoV[tiab] OR "Wuhan coronavirus"[tiab] OR SARS2[tiab] OR "SARS Virus"[Mesh] OR "SARS‐CoV‐2"[Mesh] OR "COVID‐19"[Mesh]) AND ("mental disorder"[tiab] OR "mental health"[tiab] OR "mental health program"[tiab] OR "mental health service"[tiab] OR "psychiatric service"[tiab] OR "psychological service"[tiab] OR "psychiatric health"[tiab] OR "psychological health"[tiab] OR "public health service"[tiab] OR "psychosocial intervention"[tiab] OR "psychiatric intervention"[tiab] OR "psychological intervention"[tiab] OR "psychological treatment"[tiab] OR "psychiatric treatment"[tiab] OR "psychotherapy"[tiab] OR "COVID‐19/psychology"[Mesh] OR "COVID‐19/rehabilitation"[Mesh] OR "Mental Health Services"[Mesh]) AND (Telemedicine[tiab] OR Tele‐medicine[tiab] OR Telehealth[tiab] OR Tele‐health[tiab] OR Telecare[tiab] OR "Mobile health"[tiab] OR "electronic health"[tiab] OR eHealth[tiab] OR "remote consultation"[tiab] OR remote[tiab] OR "Telemedicine"[Mesh]) AND (intervention[tiab] OR RCT[tiab] OR "controlled trial"[tiab] OR randomized[tiab] OR random[tiab] OR Randomly[tiab] OR Placebo[tiab] OR Assignment[tiab] OR "clinical trial"[tiab] OR trial[tiab] OR randomized[tiab] OR "Methods"[Mesh] OR "RCT"[Publication Type] OR "Controlled Clinical Trial"[Publication Type] OR "Placebos"[Mesh] OR "Placebo Effect"[Mesh] OR "Clinical Trial"[Publication Type] OR "Clinical Trials as Topic"[Mesh])

Embase:

(SARS:ab,ti OR 'severe acute respiratory syndrome':ab,ti OR Coronavirus:ab,ti OR 'Coronavirus infection':ab,ti OR COVID:ab,ti OR COVID‐19:ab,ti OR 2019‐nCov:ab,ti OR 2019‐nCov:ab,ti OR SARS‐Cov‐2:ab,ti OR 'Novel coronavirus':ab,ti OR 'Wuhan coronavirus':ab,ti OR SARS2:ab,ti) AND ('mental disorder':ab,ti OR 'mental health':ab,ti OR 'mental health program':ab,ti OR 'mental health service':ab,ti OR 'psychiatric service':ab,ti OR 'psychiatric health':ab,ti OR 'psychological health':ab,ti OR 'public health service':ab,ti OR 'psychosocial intervention':ab,ti OR 'psychiatric intervention':ab,ti OR 'psychological intervention':ab,ti OR 'psychological treatment':ab,ti OR 'psychiatric treatment':ab,ti OR psychotherapy:ab,ti) AND (Telemedicine:ab,ti OR Tele‐medicine:ab,ti OR Telehealth:ab,ti OR Tele‐health:ab,ti OR Telecare:ab,ti OR 'Mobile health':ab,ti OR 'electronic health':ab,ti OR 'eHealth':ab,ti OR 'remote consultation':ab,ti OR remote:ab,ti) AND (education:ab,ti OR educate:ab,ti OR school:ab,ti OR 'school based':ab,ti OR inform:ab,ti) AND (intervention:ab,ti OR 'controlled trial':ab,ti OR random:ab,ti OR randomly:ab,ti OR placebo:ab,ti OR assignment:ab,ti OR 'clinical trial':ab,ti OR trial:ab,ti OR randomized:ab,ti)

Cochrane:

ID Search

#1 (SARS):ti,ab,kw
#2 ("severe acute respiratory syndrome"):ti,ab,kw
#3 (coronavirus):ti,ab,kw
#4 ("coronavirus infection"):ti,ab,kw
#5 (COVID):ti,ab,kw
#6 (covid‐19):ti,ab,kw
#7 (2019nCoV):ti,ab,kw
#8 ("Wuhan Coronavirus"):ti,ab,kw
#9 (SARS2):ti,ab,kw
#10 MeSH descriptor: [Severe Acute Respiratory Syndrome] explode all trees
#11 MeSH descriptor: [COVID‐19] explode all trees
#12 ("mental disorder"):ti,ab,kw
#13 ("Mental Health"):ti,ab,kw
#14 ("mental health program"):ti,ab,kw
#15 ("mental health service"):ti,ab,kw
#16 ("psychiatric service"):ti,ab,kw
#17 ("psychological service"):ti,ab,kw
#18 ("psychiatric health"):ti,ab,kw
#19 ("psychological health"):ti,ab,kw
#20 ("public health service"):ti,ab,kw
#21 ("psychosocial intervention"):ti,ab,kw
#22 ("psychiatric intervention"):ti,ab,kw
#23 ("psychological intervention"):ti,ab,kw
#24 ("psychological treatment"):ti,ab,kw
#25 ("psychiatric treatment"):ti,ab,kw
#26 (psychotherapy):ti,ab,kw
#27 MeSH descriptor: [COVID‐19] explode all trees and with qualifier(s): [psychology ‐ PX]
#28 MeSH descriptor: [COVID‐19] explode all trees and with qualifier(s): [rehabilitation ‐ RH]
#29 MeSH descriptor: [Mental Health Services] explode all trees
#30 (telemedicine):ti,ab,kw
#31 (tele‐medicine):ti,ab,kw
#32 (telehelath):ti,ab,kw
#33 (tele‐health):ti,ab,kw
#34 (telecare):ti,ab,kw
#35 ("mobile health"):ti,ab,kw
#36 ("electronic health"):ti,ab,kw
#37 (ehealth):ti,ab,kw
#38 ("remote consultation"):ti,ab,kw
#39 MeSH descriptor: [Telemedicine] explode all trees
#40 MeSH descriptor: [Telerehabilitation] explode all trees
#41 #1 or #2 or #3 or #4 or #5 or #6 or #7 or #8 or #9 or #10 or #11
#42 #12 or #13 or #14 or #15 or #16 or #17 or #18 or #19 or #20 or #21 or #22 or #23 or #24 or #25 or #26 or #27 or #28 or #29
#43 #30 or #31 or #32 or #33 or #34 or #35 or #36 or #37 or #38 or #39 or #40
#44 #41 and #42 and #43
